# Comparison of Smoothing Filters’ Influence on Quality of Data Recorded with the Emotiv EPOC Flex Brain–Computer Interface Headset during Audio Stimulation

**DOI:** 10.3390/brainsci11010098

**Published:** 2021-01-13

**Authors:** Natalia Browarska, Aleksandra Kawala-Sterniuk, Jaroslaw Zygarlicki, Michal Podpora, Mariusz Pelc, Radek Martinek, Edward Jacek Gorzelańczyk

**Affiliations:** 1 Faculty of Electrical Engineering, Automatic Control and Informatics, Opole University of Technology, 45-758 Opole, Poland; natalia.browarska@gmail.com (N.B.); j.zygarlicki@po.edu.pl (J.Z.); michal.podpora@gmail.com (M.P.); m.podpora@po.edu.pl (M.P.); 2 Department of Computing and Information Systems, University of Greenwich, London SE10 9LS, UK; 3 Department of Cybernetics and Biomedical Engineering, FEECS, VSB-Technical University Ostrava, 708 00 Ostrava-Poruba, Czech Republic; radek.martinek@vsb.cz; 4 Department of Theoretical Basis of BioMedical Sciences and Medical Informatics, Nicolaus Copernicus University, Collegium Medicum, 85-067 Bydgoszcz, Poland; medsystem@medsystem.com.pl; 5 Institute of Philosophy, Kazimierz Wielki University, 85-092 Bydgoszcz, Poland; 6 Outpatient Addiction Treatment, Babinski Specialist Psychiatric Healthcare Center, 91-229 Lodz, Poland

**Keywords:** electroencephalography, Brain-Computer Interfaces, digital filtering, Emotiv Flex, signal processing

## Abstract

Off-the-shelf, consumer-grade EEG equipment is nowadays becoming the first-choice equipment for many scientists when it comes to recording brain waves for research purposes. On one hand, this is perfectly understandable due to its availability and relatively low cost (especially in comparison to some clinical-level EEG devices), but, on the other hand, quality of the recorded signals is gradually increasing and reaching levels that were offered just a few years ago by much more expensive devices used in medicine for diagnostic purposes. In many cases, a well-designed filter and/or a well-thought signal acquisition method improve the signal quality to the level that it becomes good enough to become subject of further analysis allowing to formulate some valid scientific theories and draw far-fetched conclusions related to human brain operation. In this paper, we propose a smoothing filter based upon the Savitzky–Golay filter for the purpose of EEG signal filtering. Additionally, we provide a summary and comparison of the applied filter to some other approaches to EEG data filtering. All the analyzed signals were acquired from subjects performing visually involving high-concentration tasks with audio stimuli using Emotiv EPOC Flex equipment.

## 1. Introduction

The investigation of brain waves with the implementation of the EEG (electroencephalography) data is very useful, as it can help understanding human mental condition such as stress, burnout exhaustion, or mental fatigue [1,2,3,4,5]. It can also provide information regarding the health condition of the tested subjects without the need of surgical intervention [6]. This is because electroencephalography is a noninvasive medical technique introduced in 1875 by Richard Caton [1].

The authors of this work (as a part of a larger project) carried out a series of experiments with the implementation of an inexpensive EEG headset—Emotiv EPOC Flex [7,8]. The quality of the data acquired using this headset strongly differs from those obtained from the clinical amplifiers. The experiments were initially performed in order to check the influence of various sounds (audio stimuli) on human concentration capabilities during performance of various visually stimulated tasks.vData obtained for this study purpose is of particular character, as the Emotiv headsets usually preprocesses the recorded data, which results with some information removal from the obtained signal [7,9]. The device uses 5-th order Sinc filter—0.5–45 Hz and Notch filters (at 50 and 60 Hz) for the power disturbances removal [7].

In [10], the authors focused on design, development, and testing of various smoothing filters of clinical quality data. For these study purposes they decided to implement similar filters, but on data recorded with the customer-grade, inexpensive, off-the-shelf BCI-headset—Emotiv EPOC Flex. As it was mentioned above, the obtained data quality differs from the one from clinical signals, which affected the choice of the applied filtering. Implementation of smoothing filtering usually positively affects the quality of EEG data, as it makes them more legible for potential diagnostic purposes and does not affect their content [10,11,12].

### 1.1. Study Background

It is claimed that the human brain is the most complicated human organ and can be compared to a very powerful and complex supercomputer [13,14,15]. Until today, no one was able to recreate and simulate successfully its entire structure. Nowadays, both medicine and information technology are dynamically developed. One of the exemplary results of this collaboration are Brain–Computer Interfaces (BCIs), especially those based on electroencephalography examination. The main aim for implementation of the BCI systems is to entirely replace muscle engagement for controlling external devices, such as computers, intelligent home systems, computer games, and applications, or to support solutions for people using wheelchairs, neuroprostheses, speech prosthesis, and exoskeletons [9,15,16,17,18].

A significant number of people listen to music during everyday activities while driving a car, doing sports, working, studying, and leisure time. It is a good way to enhance listeners’ mood, to relax, reduce stress, relieve tension, or even increase focus [19]. It is clearly visible at the physiological system of the human body at the frequencies of theta and alpha [20]. Moreover, background music is commonly used in public spots, such as restaurants, hotels, offices shops, banks, or shops [21,22,23]. It affects people’s behavior, in particular their concentration level while doing various tasks. This is one of the reasons why we decided to carry out a series of experiments, where participants were influenced with the audio stimuli.

A natural consequence of the experiments’ range was selection of the method—we decided to use electroencephalography (EEG) as a noninvasive method of signal acquisition [9,15,17]. It enables measurement of field potential in the space around neurons, which results in recording of the electrical activity of the brain [15,16,17]. The appropriate electrodes location on the scalp surface plays a crucial role in the signal acquisition process. The EEG channels are placed on the scalp surface in accordance with the 10–20 system [15]. The EEG signal can be characterized with a high time activity about 1 [ms], also its amplitude and frequency are inconstant. Moreover, the EEG waveform is never a simple harmonic signal [15,17].

The most popular method for the EEG frequency ranges classification is the use of Greek letters: Alpha (α—8–12 Hz), Beta (β—13–30 Hz), Delta (δ—<4 Hz), Gamma (γ—>30 Hz), Theta (θ—4–7 Hz), and Mu—similar to alpha (μ—8–12 Hz, which strictly depend on the activity the brain performance [3,8,9,11,24,25,26].

The above-mentioned frequency ranges reflect the rhythmic activity and state of the brain and are linked to various functions such as motor, sensory, or cognition [8]. The lowest rhythm—delta—occurs in a deep sleep state and it is also typical for brain damage in case of young people [27,28]. The theta waves relate to intense emotions, dreams, and meditation [28,29,30,31]. The alpha waves are associated with relaxation (especially with eyes closed). The beta rhythm is typical for everyday activities but it can be also related to the influence of certain drugs, such as diazepam and zolpidem [32,33]. Finally, the gamma waves are associated with awareness, problem-solving, and perception [28].

It is important to mention that it is possible to find significant differences in EEG recordings in relation with the patients’ emotional intelligence, where high emotional intelligent individuals showed less desynchronization in the upper alpha band and more left hemispheric theta desynchronization [30,31,34].

### 1.2. Signal Processing of EEG Data

The electroencephalogram data is a result of electrical activity of brain measurement [11,15,16]. It has three important features which determine the process of its acquisition and analysis:

- low amplitude,
- strong non-stationary character, and
- narrow range of frequency band.

The analog signal is acquired using noninvasive EEG sensors placed on the human scalp. Its amplitude is approximately several dozen micro-volts, which enforce signal quantization and discretization on the level of several thousand (V/V) [35]. Due to the low amplitude level, recording the EEG signal is a difficulty [11]. Furthermore, a relatively small, electromagnetic interference near the electroencephalogram affects the signal. Thus, signal conditioning systems in EEG-recording devices are equipped with additional systems, which eliminate distortions during the filtering process. This method might, however, distort and/or exclude diagnostic information from the analyzed signal. It is clearly visible, in particular with the application of the inexpensive consumer-grade devices. The interference frequently occurs in the recorded EEG signal and may cause significant problems with the data interpretation [10,11,15,16].

The strong non-stationary character of the EEG signal makes the overall data analysis a very challenging task [9,10,15]. Therefore, it can be useful to apply various signal processing methods such as the well-known Fourier transform, which averages the spectrum of the signal. Additionally, it is possible to observe a spectrum blur effect due to the lack of synchronization of the analyzed signal’s components, which complicates the process of proper analysis of the EEG data. For this reason, a very popular, although also a very simple, Fourier transform is not frequently applied for the purpose of the EEG signals’ analysis. More popular method is a short-term Fourier transform (STFTT), where duration of the time-shifting analysis window is minimized, which results in a loss of resolution in the frequency domain. However, the effect of blurring and averaging the spectrum is still visible; thus, the research and application of alternative methods of EEG signal analysis in the time-frequency domain, such as wavelet analysis and/or Cohen-class transformations are more applicable [11,35].

The third feature of the EEG signal is the narrow frequency band. It is advantageous for the development of EEG signal acquisition systems. The bandwidth lower than the basic harmonic frequency of the power network signal (50 Hz in Europe, 60 Hz in the United States) allows effective filtering of source interference.

## 2. Materials and Methods

**Figure 1:**
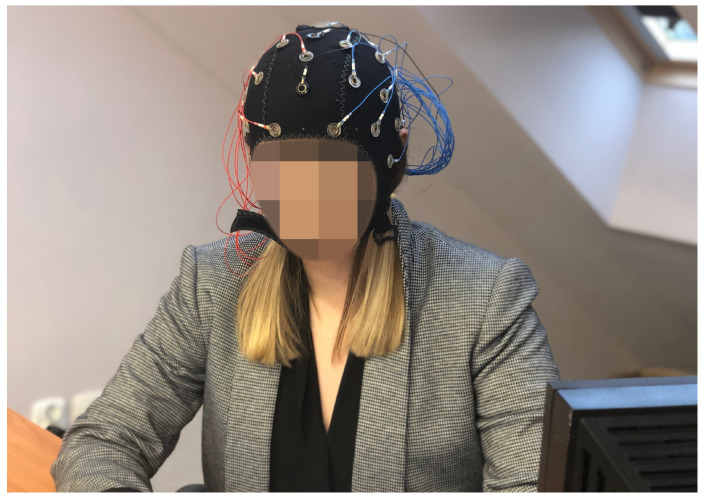
Emotiv EPOC Flex device [7].

For this study purposes, the authors of this work decided to carry out all measurements with the implementation of the Emotiv EPOC Flex device with gel-based sensors (illustrated with Figure 1). The EPOC Flex is a 32 channel EEG-based Brain–Computer Interface. It is an inexpensive, consumer-grade, high market availability EEG device [7]. It delivers configurable channels location on a standard 32 channel 10–20 map and 0.51 μV EEG Resolution. This customer-grade EEG with “Ag” sensors allows recording signals and is mainly dedicated to academic research, however, these signals are not totally raw, but preprocessed and do not contain full information [7,15]. Some sources, however, claimed Emotiv Flex to provide clinical quality of data [36].

**Figure 2:**
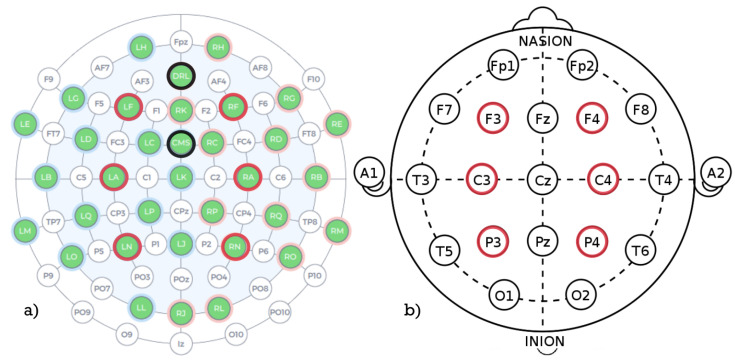
Schema of the sensors arrangement: (**a**) Emotiv Flex default electrodes arrangement; (**b**) electrodes chosen for analysis.

For this study’s purposes, the electrodes were placed in accordance with the 10–20 system in locations illustrated with Figure 2. For this study’s purposes 32 sensors were applied: (Cz, Fz, Fp1, F7, F3, FC1, C3, FC5, FT9, T7, TP9, CP5, CP1, P3, P7, O1, Pz, Oz, O2, P8, P4, CP2, CP6, TP10, FC6, C4, FC2, F4, F8, and Fp2)—marked with a green color in Figure 2, where the referencing electrodes were marked with black [7]. The authors of this paper decided to focus on analysis of data obtained from six channels only: F3, F4, C3, C4, P3, and P4 marked with the red color in Figure 2.

The choice for a limited amount of electrodes has been done carefully, based on not only authors’ professional experience, but also on thorough literature study. As it was mentioned above, analysis of biomedical data, in particular EEG, is a very challenging task [9,10,15,35]. Having a large number of EEG channels makes the whole data analysis process more complex, which can be eased by inter alia selection of only those necessary channels [37,38,39,40]. It also allows reducing the set up time [37,41]. Moreover, using less channels enables to make the BCI systems more compact [38]. Very limited number of channels (only two) is applied in inter alia polysomnography studies, where EEG data is one of the analysed. So far, such an amount of electrodes seems to be enough for research or medical purposes [41]. The use of six channels only in BCI applications can be found in numerous studies and it has been proven to provide similar to the expanded channel sets performance [42,43,44,45,46]. In [43] 6-channel system was compared to the one with 19 electrodes for the benefit of a system with fewer electrodes. The authors of this work decided to choose symmetrically-located electrodes in order to cover frontal, central and paternal lobes.

### 2.1. Carried out Experiments

For this study’s purposes, a series of experiments was carried out and will be in detail presented in further part of this work. Key information regarding patients was taken into account such as age, gender, physical, and mental condition [25]. The purpose of the experiment was to examine the influence of ambient music—an audio stimulation, during logical task performance—simply ERP visual task—word search. The authors prepared three scenarios in order to test the impact of selected sounds on the examined group:

- without an audio stimulation,
- with distracting sounds, and
- with concentration stimulating sounds.

Each of the three tasks was preceded by the ambient sounds of nature due to the expected relaxing/calm state before the next phase. For each test stimuli was shown to the patient at the same time, while the EEG data was recorded. A detailed scenario is described in the Table 1.

Classifications of cognitive and emotional states based on bio-signals have been analyzed in many scientific papers, in particular regarding analysis of EEG data (see, inter alia, in [11,47,48]) and is planned to be expanded in future work of authors of the hereof paper.

There are many different types of devices, which enable recording of EEG signals and brain activities. These devices differ in their design, price, and availability, but mainly in the type of applied and the number of electrodes they have [2,11]. It is also medically proven that different parts of the brain are responsible for controlling specific activities, actions, or mental states [2,15].

**Figure 3:**
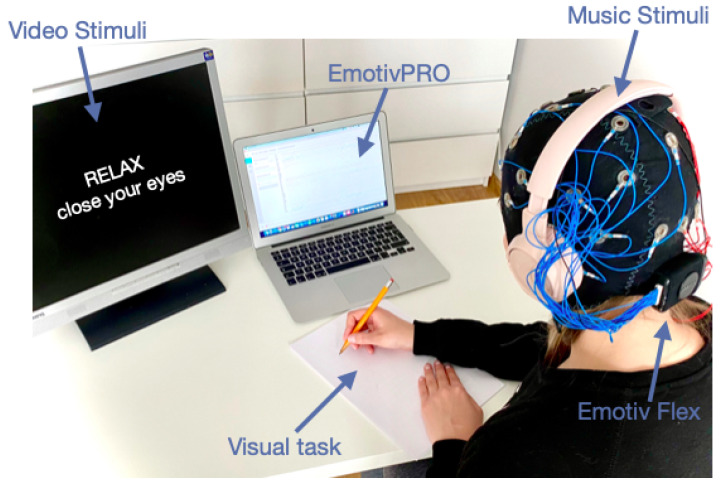
Stimuli workstation.

The experiments were carried out in the laboratory environment. The stimuli view was displayed on an LCD monitor (19-inch, BENQ) with a 60 Hz refreshing rate. Stereo headphones (JBL) were used and the sound volume was set at a fixed and comfortable level. The participants watched and listened to 30 min of audio-video material. The workstation is shown in Figure 3. The experiment lasted for approximately 50 min including 20 min for Emotiv Flex setup, in accordance with the scenario presented in Table 1.

During the experiment, patients were doing logical exercises, such as easy crosswords. The purpose of this test, prepared by authors, was to trigger the patient’s concentration. It was also attempted to check the accuracy of the solved test. Ten participants (2 females; 8 males) with an age average of 34.8 (from 22 to 48 years) participated in the experiment as volunteers. All of them fulfilled following exclusion criteria:

- report of any physical or mental health problems,
- use any medication, and
- problems with sleep.

The participants were among the members of research team and have given their oral and written consents to take part in this study, see Table 2):

1. age,
2. gender,
3. drugs taken, and
4. their current mood (mental condition) in 0 to 10 scale.

### 2.2. Performed Data Analysis

**Figure 4:**
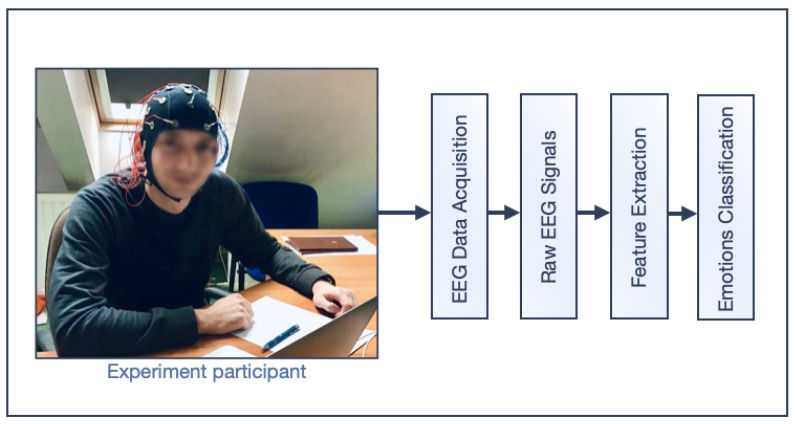
Data analysis scenario.

As it was mentioned above, the study was carried out with the participation of ten healthy volunteers in accordance with the scheme illustrated with the Figure 4.

In order to make the obtained data more legible the authors decided to apply and compare various smoothing filters. As the data is initially preprocessed by the device itself (0.5–45 Hz, 5-th order Sinc filter) it was very challenging to choose appropriate filters, as the data for analysis did not contain full information. It was not raw, clinical-level data. Due to the blinking artifact, the data was filtered with a high-pass 4-th order Butterworth filter, with the cut-off frequency of 4 Hz. The very first step involved baseline removal [49]. For this study’s purposes, various smoothing filters were applied at the initial stage in order to improve the quality of the analyzed data. As the data have already been preprocessed during recording, they did not contain full information.

The whole signal processing procedure was carried out in Matlab 2017b software (Curve Fitting Toolbox, Signal Processing Toolbox, DSP System Toolbox) from MathWorks (Natick, MA, USA). The analyzed data were recorded from the six following locations: F3, F4, C3, C4, P3, and P4, which represent the following locations of the brain: “frontal”, “central”, and “peripheral”, which were chosen by the authors of this work based on their wide research experience as the most optimal for this study purposes.

Based on authors’ previous experience with clinical data and smoothing filtering, which was in detail described in [10], it was attempted to apply similar filters (same parameters) but on data that did not contain full information (as it was mentioned above). Smoothing filtering has a wide applicability in analysis of EEG data, which can be seen in [11,50,51,52,53,54]. The authors of this work applied five different smoothing filters, including the following [10]:

- Filter 1—classic moving average “smooth” filter with defined by default smoothing parameter (span) set to 5,
- Filter 2—classic moving average “smooth” filter with defined smoothing parameter (span) set to 15,
- Filter 3—classic moving average “smooth” filter of the 2nd order, which uses Savitzky–Golay filter as a method,
- Filter 4—9th order one-dimensional median filter,
- Filter 5—Savitzky–Golay FIR smoothing filter of the 4th order and 27 frame length.

As it was described in detail in [10] the “smooth” filter is a classic moving average filter, which works in a way that it replaces each data point with the average of the neighbor data points. Its work is similar to the low-pass filtering [10,35]. Moreover, it smooths the data in the column vector *y* using a moving average filter. Results are returned in the column vector *yy*, as shown with the (1) and (2). The default span for the moving average, as it was mentioned above, is 5 (for Filter 1) and the defined one (for Filter 2–15).

(1) yy=smooth(y);

Furthermore, the first few elements of the above mentioned yy can be given by

(2) $$\begin{array}{c} yy(1)=y(1)\\ yy(2)=(y(1)+y(2)+y(3))/3\\ yy(3)=(y(1)+y(2)+y(3)+y(4)+y(5))/5\\ yy(4)=(y(2)+y(3)+y(4)+y(5)+y(6))/5 \end{array}$$

The applied Filter 3 is also a moving average filter, however, this time its coefficients were determined with an unweighted linear least-squares regression and a polynomial model of specified degree, which is default 2. It is the simplest Savitzky–Golay filter.

The median filter, which was applied as Filter 4 is a nonlinear, one-dimensional filter, where the mean value of a sequence (of values in the ascending order of data) of the processed point and its surroundings is measured. The output signal consists of the individual median values of all the windows applied. The chosen order of the filter was 9 [10,55].

The last one tested filter (Filter 5) was Savitzky–Golay filter, which is a least-square digital polynomial filter, which smooths the fluctuations and increases the signal–noise ratio (SNR) without significantly distorting the data [10,56]. It is a Finite Impulse Response (FIR) filter, where its order must be less than frame length (b=sgolay(order,framelen)). In the case of (3)

(3) order=framelen−1

no smoothing is carried out. The applied parameters for the Filter 5 were as follows [10],

- order: 4th and
- frame length (*framelen*): 27.

## 3. Results

**Figure 5:**
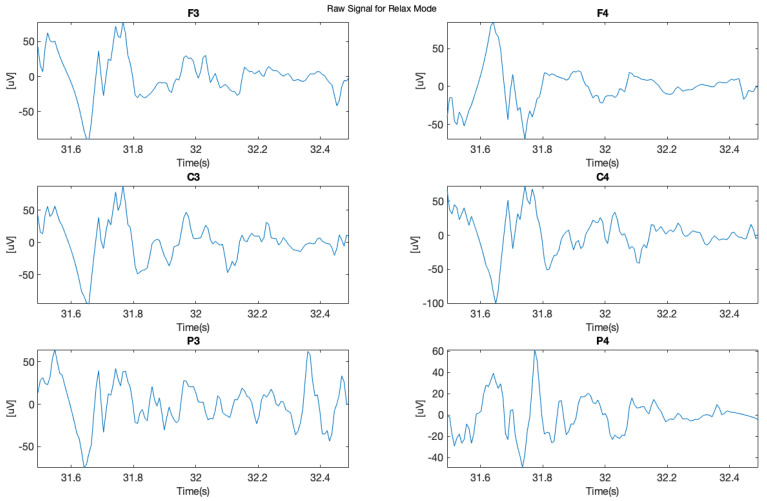
Raw signal during relax.

In this study, we chose two signal samples in order to perform further analysis. The first sample (Figure 5 shows raw signal recorded during relax mode for each of the applied six electrodes F3, F4, C3, C4, P3, and P4), where it is possible to see clear “alpha” wave on the 1 s sample.

**Figure 6:**
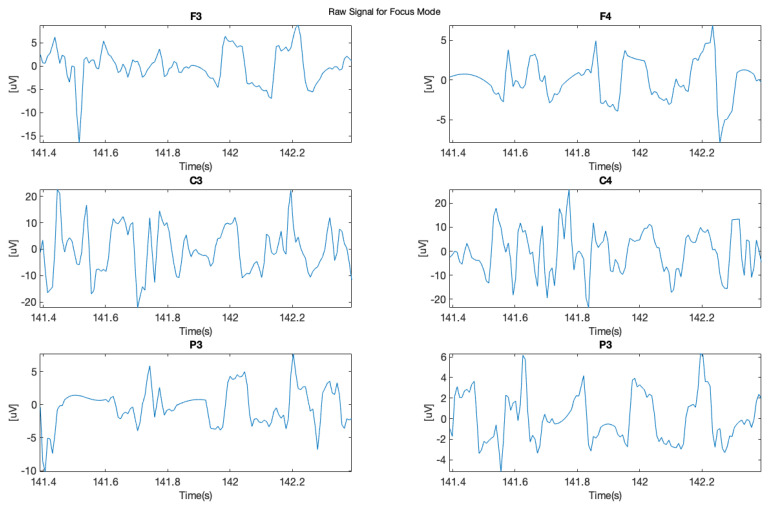
Raw signal—focused subject.

The next phase of this experiment involved focusing on mental tasks when 14 Hz stimulating sound was applied. In Figure 6, it is possible to observe in the data from the same subject, recorded during this phase, significant density of the brainwaves, which corresponds with the higher cognitive brain functioning and with clearly visible “beta” waves. The frequency of the above mentioned 14 Hz audio stimulation was chosen to force the brain to work in the beta range of the lower low-beta band (12–15 Hz). It was also the lowest limit frequency of the audio path of the applied sound-set.

**Figure 7:**
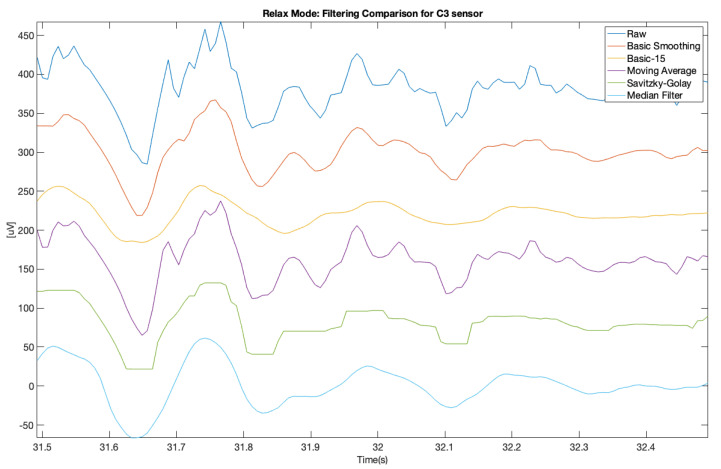
Filter comparison—relax mode (C3 channel).

**Figure 8:**
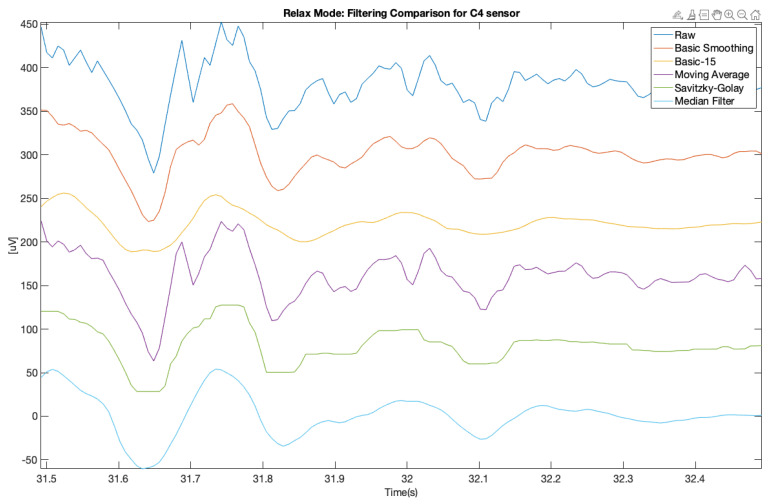
Filter comparison—relax mode (C4 channel).

In order to improve the data quality, we applied various smoothing filters, which were also applied in another study [10] (on different types of data from a clinical setting). The applied filters were also described above. The presented samples are for the electrodes C3 and C4, which represents central location on the scalp. The Figure 7 and Figure 8 show a comparison of the raw signal and the five applied smoothing filters for the relax mode.

**Figure 9:**
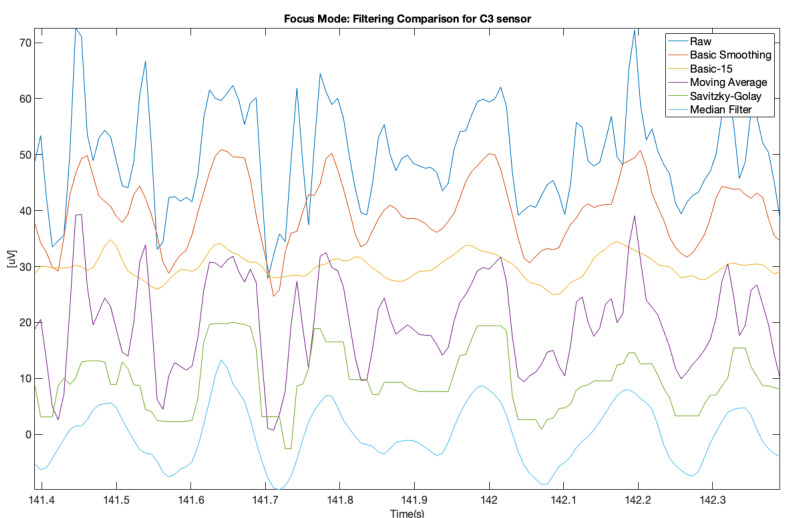
Filter comparison—focus mode (C3 channel).

**Figure 10:**
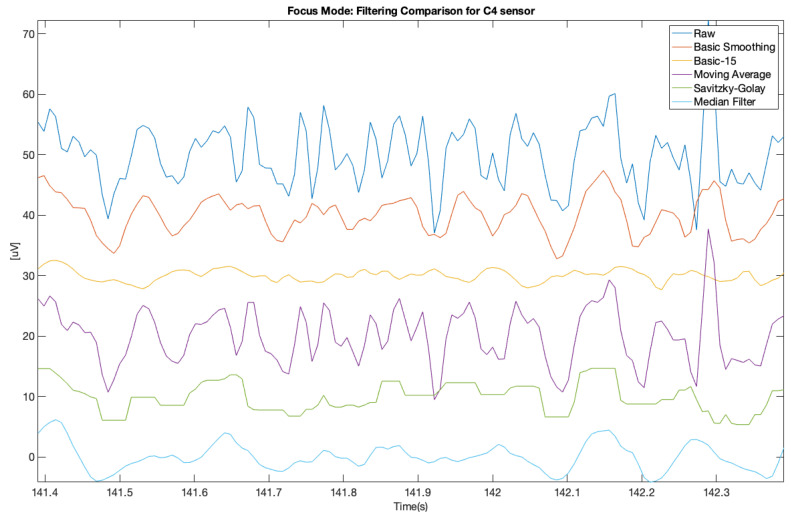
Filter comparison—focus mode (C4 channel).

The same was done for the electrodes C3 and C4 for the focus mode (see Figure 9 and Figure 10).

**Figure 11:**
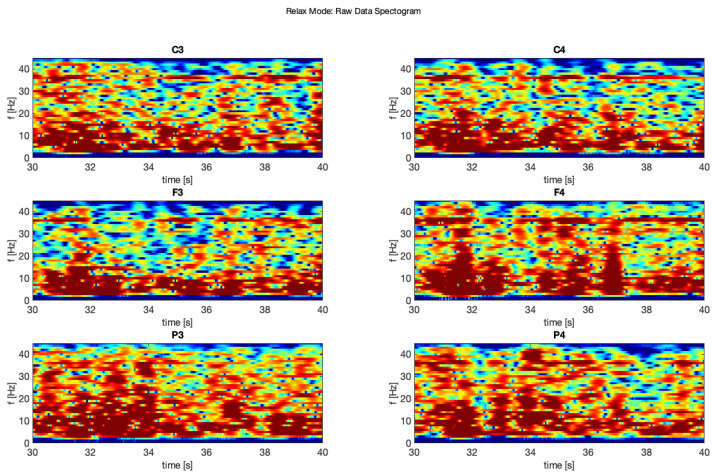
Raw signal—relax.

**Figure 12:**
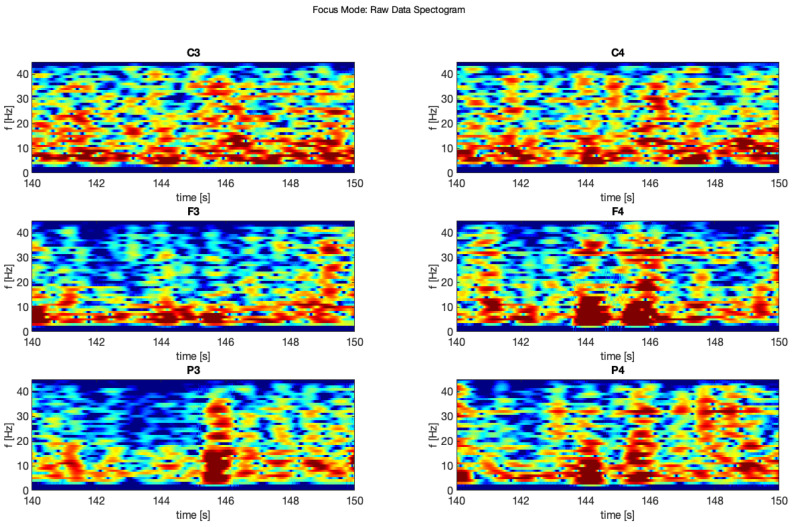
Raw signal—focus.

**Figure 13:**
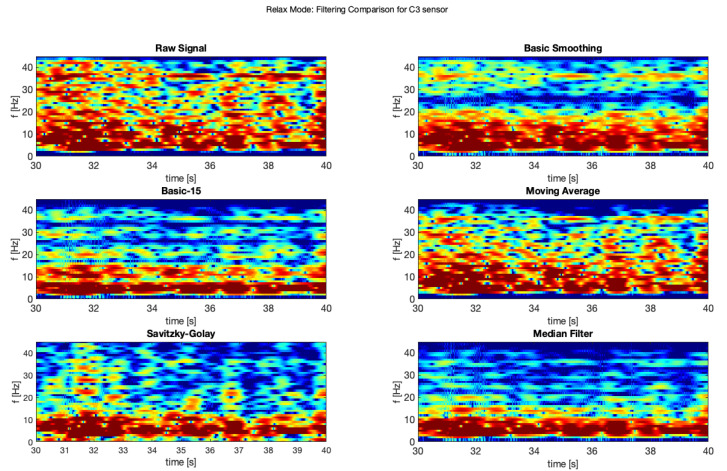
Filter comparison—relax (C3).

**Figure 14:**
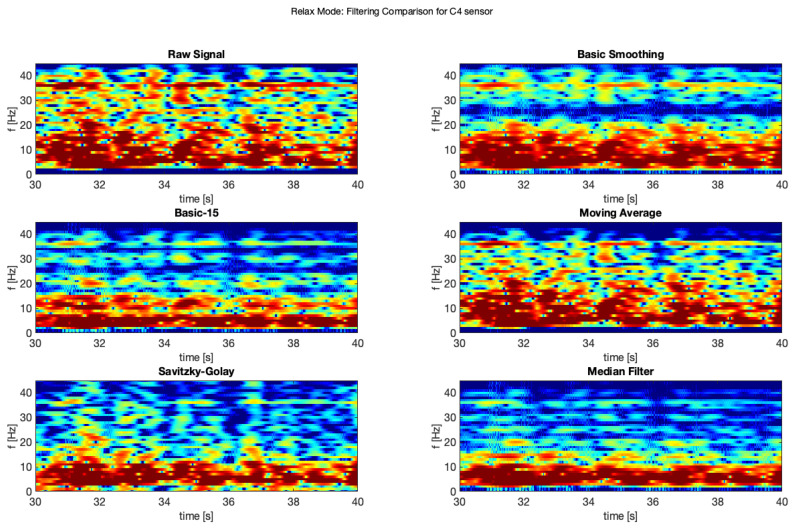
Filter comparison—relax (C4).

**Figure 15:**
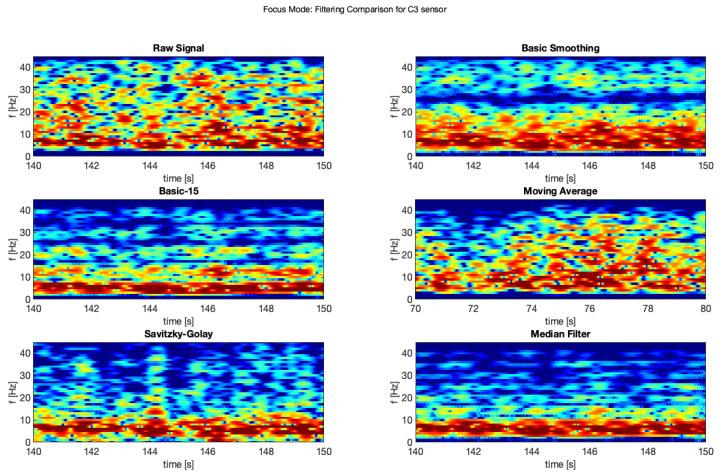
Filter comparison—focus (C3).

**Figure 16:**
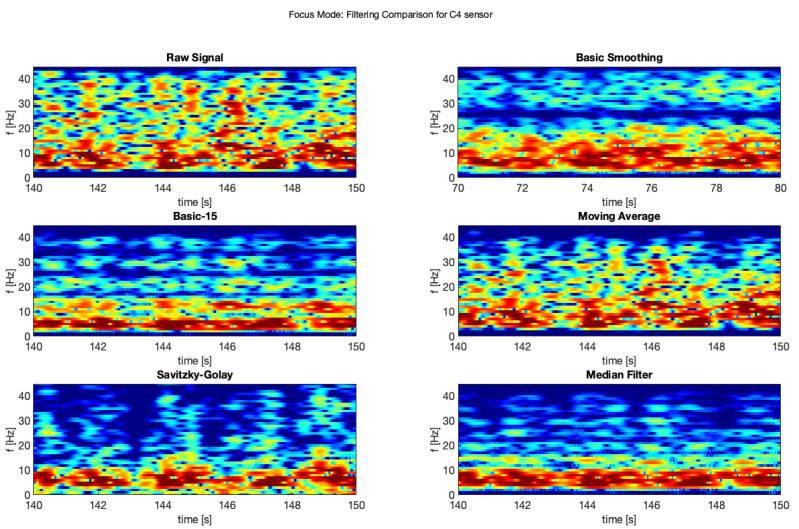
Filter comparison—focus (C4).

It is clearly visible that the 3rd filter (moving average) is the proper one for this purpose, unlike in [10]. Other filters removed too much crucial information from these samples and flattered the characteristic “peaks” in the EEG signal. Similar situation is visible at 10 s sample on spectrograms (Figure 11, Figure 12, Figure 13, Figure 14, Figure 15 and Figure 16). At first it was presented in Figure 11 and Figure 12.

Below was presented a comparison of raw signal and five smoothing filters (see Figure 13 and Figure 14) for electrodes C3 and C4 recorded during relax mode.

Moreover, the same comparison for the electrodes C3 and C4 for the focus mode (Figure 15 and Figure 16).

Due to the fact that visual estimation of signal filtering quality is insufficient, the authors have decided to implement the filtering efficiency rating criteria based on pointing characteristic “peaks” of the EEG signal.

The digital input signal, sample by sample, was sent to the First In First Out (FIFO) queue, in which the values of the three consecutive samples with the numbers 1, 2, and 3 were compared. In case the value of sample 2 is greater than the value of samples 1 and 3, the time instant at which the sample 2 occurred is remembered as the time of the new peak occurrence and the value of sample 2 becomes new peak provided that the time interval between the current peak and the previous peak satisfies the frequency condition. The frequency condition is defined by the sampling frequency of the analyzed signal *fs* and the upper cut-off frequency of the band of the recorded EEG signal—*fg*. The signal sampling frequency was 128 Hz and the upper limit frequency of the signal band was 30 Hz. The frequency condition here defines the minimum distance expressed in samples between the recorded peaks on the basis of the relationship: (4). All analyzed EEG wave-forms were tested on the basis of the presented algorithm.

(4) $$w=round\frac{fs}{fg}-1=round\frac{128\;\mathrm{Hz}}{30\;\mathrm{Hz}}=3$$

Figure 17, Figure 18, Figure 19 and Figure 20 (same—1 s samples as on Figure 7, Figure 8, Figure 9 and Figure 10) was shown visually how this script works. This method also confirms that moving average filter (Filter 3) gave the best results.

In order to make the obtained results more reliable, we decided to compare the same 10 s samples as those illustrated with the spectrograms (Figure 13, Figure 14, Figure 15 and Figure 16) both for relax and focus modes for channels C3 and C4 and counted every “peak” on this signal samples for raw and filtered signals.

For this purpose, the below Equation (5) can be applied,

(5) $$FeatureCoverage=\frac{PeaksFilteredSignal}{PeaksRawSignal}\;*100\%$$

**Figure 21:**
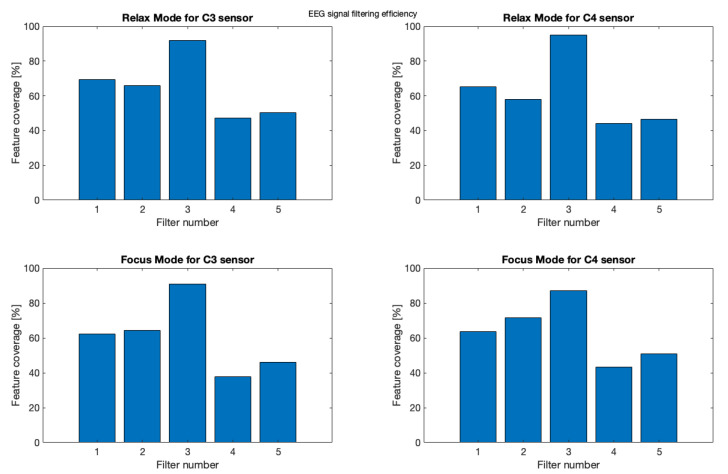
Filtering Comparison—percentage feature coverage.

Results of this filter comparison were shown on bar graphs illustrated with the Figure 21.

This study confirms the best compatibility of applying moving average filter (Filter 3) with average peak coverage for every sample—91.14%. Second best result achieved basic smoothing filter (Filter 1)—65.16% and the third was Filter 2 with the score of 64.92% of feature coverage. The Filter 5 gained 48.42% and Filter 4 gave the worse result of 43.06% as shown in Table 3.

## 4. Discussion

The data were recorded with the Emotiv EPOC Flex EEG headset and the sampling rate was only 128 Hz. It is typical for all Emotiv products to generate “spikes” visible in the time-domain figures. It also happened during previous studies on data recorded with the implementation of Emotiv EPOC EEG [9,15]. This was also a reason for decision on using smoothing filtering. Due to the fact that the data has already been pre-processed during recording—it did not contain full information.

One of the key factors leading the authors to this research was is the great popularity of the Emotiv EPOC Flex headset (as well as other “consumer-grade” products) among young researchers, which tend to believe that the acquired signal is reliable. There is not only a need to remind them that the signal is already preprocessed when they believe it is “raw” data, but also remind them that this equipment does not offer “clinical quality” EEG data [57,58,59].

The filters applied in this work seem to be basic, but they are not only off-the-shelf filters. All the filters were chosen carefully, their parameters were thoroughly adjusted after performance of numerous studies (see in [10,60] and tests. Implementation of similar (but not the same) smoothing filters for the purpose of analysis of biomedical data can be found in inter alia the following positions (which are very recent) [61,62,63,64]. A little bit similar study, where only Savitzky–Golay filters (but with different parameters) were applied was described in [65], but no other smoothing filters were neither applied nor compared. It is because in most of the literature positions comparison of various types of smoothing filters is not present and they focus mainly on one filter type, at most—Savitzky-Golay. Furthermore, the choice of filtering parameters (window and frame) differs from the one used by the authors of this work.

Previous studies of some of the authors of this work (see in [10]) proved that using smoothing filters can significantly improve the legibility of the analyzed data and the most promising results were obtained while using Savitzky–Golay filters. In this case, however, the best results are obtained using moving average (Filter 3), where the coverage spikes accuracy was 91.14%. It is probably because of the data quality and applied equipment (Emotiv EPOC Flex), which is less precised than clinical appliances.

It is also important to state that processing EEG data acquired with two different hardware devices (clinical- and consumer-grade) can lead to different results. For this reason the authors have investigated the application of smoothing filters to “clinical quality” EEG data in one publication (see in [10]), and for this study purposes they decided to investigate the application of smoothing filters to “consumer-grade” hardware-acquired EEG data. The authors’ intention was to pinpoint that the “consumer-grade” equipment should be considered separately, and the pursuit of the optimal smoothing filter for an application should be done wisely and while remembering that the “consumer-grade” equipment does not provide “real” raw data [58,66].

It is important to mention that the very first aim of this work was the acquisition of the applied data set to distinguish appropriate EEG signals’ features during audio stimulation tasks and then a comparison of 3 different mental states, depending on subjects’ alertness, affected with different ambiance sounds. We also wanted to develop a method for the “focus” state feature extraction using artificial intelligence algorithms; however, when starting the data preprocessing using smoothing filters applied in our previous analyses on EEG clinical data, the results were unsatisfactory. Thus, we realized that there is a significant difference between filtering the signal acquired using the high-quality clinical appliance and the one with the low-cost consumer-grade BCIs [10,67].

Based on our previous experiences and on the findings in this work, we have decided to carry out a comparison of smoothing filters on signals recorded from the Emotiv EPOC Flex device in order to make further signal analysis reliable and efficient. We have also decided to create a novel criteria method which compares the number of characteristic “peaks” in the analyzed EEG signal after filter application.

## 5. Conclusions and Future Work

To sum it all up, the thorough literature study did not find any papers regarding comparison of the smoothing filters (various types) the way this has been done in this paper. We were strongly motivated to find an efficient and useful smoothing filter (not only for processing medical-quality EEG data, but also for the data provided by a cheaper consumer-grade EEG hardware). We have evaluated some of the popular filtering approaches as well as we have included a smoothing filter implemented using the Savitzky–Golay filter. While the thorough literature study did not find any papers that would compare various types of smoothing filters for EEG data the way this has been done in this paper, we decided to share the results of this research. The comparison was presented within the paper and depicted in numerous easy-to-interpret figures. The novelty of the research presented in this paper and the proposed approach lies on

1. the parameters of the filters chosen for good performance when using on EEG data and
2. testing and comparison of various types of smoothing filters.

Issues discussed in this paper have great potential for further research, which is going to be thoroughly expanded in the near future. We are also working on a method for reducing EEG signal interference—shielding hardware, when using consumer-grade BCIs. Audio stimulation can have strong impact on the examined EEG signal. Those methods can also help to explain the role of cortico-subcortical loops not only in the perspective of physiological control of the majority of sensory-cognitive, emotional, and psycho-motor functions, but also in the explanation of mental disease pathophysiology and the brain structures of the mind [68].

Further research plans include study protocol improvement, which—based on authors’ experience—was too long and might have caused some frustrations among subjects participating in this study (see in [15]).

As of now, the evaluation of the tested methods is mostly based on authors’ expertise and long-time experience with bio-signals acquisition and processing, it is planned (and currently at the implementation stage) to apply expert methods based on policy-based computing (this also includes fuzzy logic reasoning) [69,70,71,72,73,74].

The next study will include simultaneous testing of patients’ attention using proper methods, such as the Wisconsin Card Sorting Test (WCST) [75], Iowa Gambling Task (IGT) [76], or Stroop Test [77]. For this reason it is necessary to involve some experienced medical professionals (e.g., psychiatrists) in order to discuss the obtained results.

Last but not least, we also would like to develop real-time systems based on noninvasive consumer-grade BCI devices. In this field, the application of the proper method for biomedical data filtering and processing will be necessary, as well as neural networks application—especially pattern recognition algorithms for mental state/emotion recognition. Thorough literature background is being carried out [78,79].

## Figures and Tables

**Figure 17:**
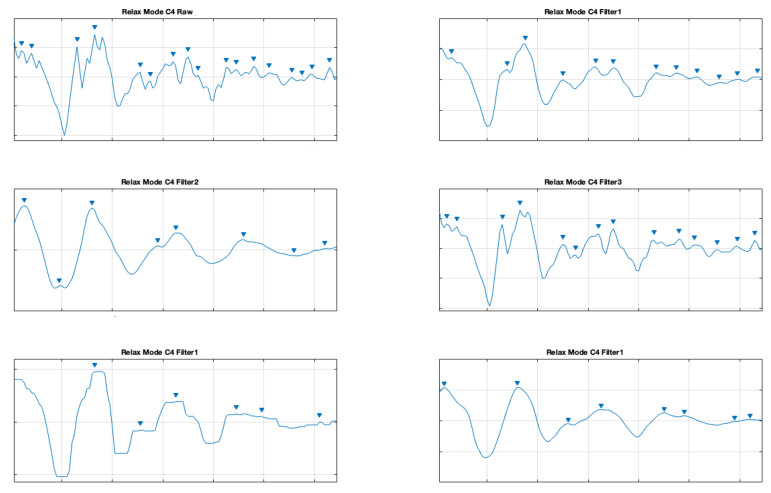
Filter comparison—relax (C3)—characteristic “peak” features.

**Figure 18:**
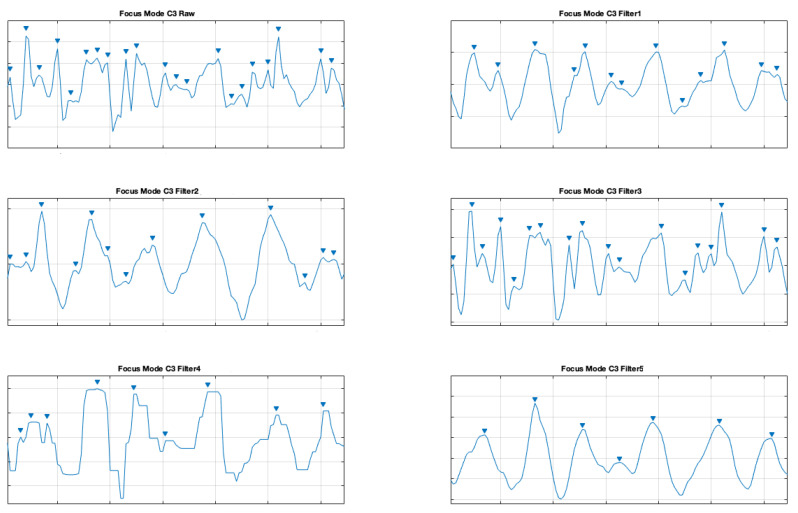
Filter comparison—relax (C4)—characteristic “peak” features.

**Figure 19:**
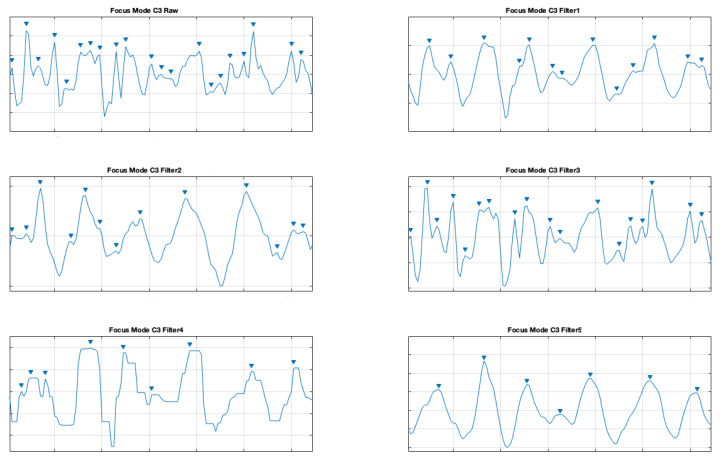
Filter comparison—focus (C3)—characteristic “peak” features.

**Figure 20:**
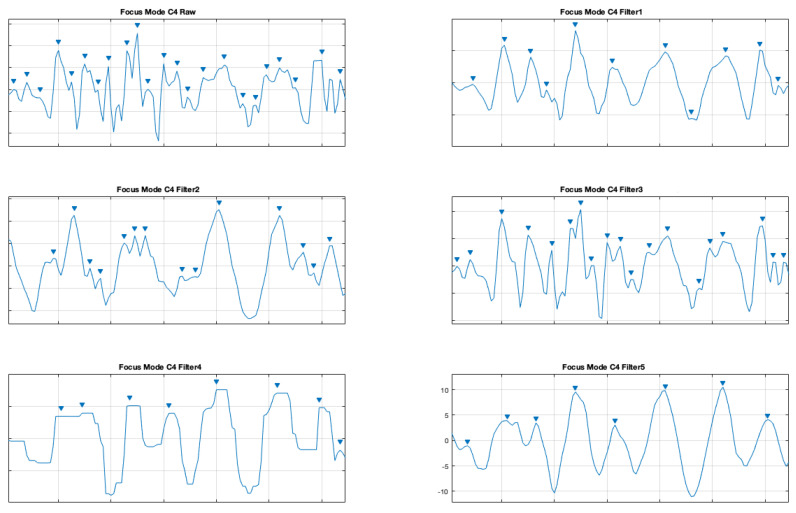
Filter comparison—focus(C4)—characteristic “peak” features.

**Table 1:** Stimuli scenario.

No.	Task	Duration	Description
1	Device configuration	20 min	General rules discussion. Cap and electrodes placement.
2	Relax	3 min	Relax with eyes closed. Relaxing sounds stimuli (nature, waves, etc.)
3	Focus	7 min	Solving easy visual task (word search) without any audio stimulation.
4	Relax	3 min	Relax with eyes closed. Relaxing sounds stimuli (nature, waves, etc.)
5	Focus	7 min	Solving easy visual task (word search) with distraction sounds of electric drill, baby crying.
6	Relax	3 min	Relax with eyes closed. Relaxing sounds stimuli (nature, waves, etc.)
7	Focus	7 min	Solving easy visual task (word search) with stimuli 14 Hz sounds.

**Table 2:** Metadata.

No.	Age	Gender	Drugs Taken	Current Mood (0–10)
1	26	f	no	7
2	30	m	no	2
3	22	m	no	7
4	48	m	no	6
5	35	m	no	4
6	42	m	no	5
7	38	m	no	6
8	30	m	no	10
9	41	m	no	4
10	36	f	no	2

**Table 3:** Filtering comparison—feature coverage accuracy.

Filter Number	Peak Coverage Accuracy %
Filter 1	65.16
Filter 2	64.92
Filter 3	91.14
Filter 4	43.06
Filter 5	48.42

## Data Availability

Data available upon request.

## Abbreviations

The following abbreviations are used in this manuscript:

BCI	Brain–Computer Interfaces
EEG	electroencephalography
DSP	digital signal processing
CNS	central nervous system
S-G	Savitzky–Golay filter
WCST	Wisconsin Card Sorting Test
IGT	Iowa Gambling Task
FIFO	First In, First Out
